# Evaluating the performance of the Breast and Ovarian Analysis of Disease Incidence Algorithm model in predicting 10-year breast cancer risks in UK Biobank

**DOI:** 10.1093/jnci/djae335

**Published:** 2024-12-12

**Authors:** Carmen Petitjean, Naomi Wilcox, Lorenzo Ficorella, Joe Dennis, Jonathan Tyrer, Michael Lush, Jacques Simard, Douglas Easton, Antonis C Antoniou, Xin Yang

**Affiliations:** British Heart Foundation Cardiovascular Epidemiology Unit, Department of Public Health and Primary Care, University of Cambridge, Cambridge CB2 0BB, United Kingdom; Victor Phillip Dahdaleh Heart and Lung Research Institute, University of Cambridge, Cambridge CB2 0BB, United Kingdom; Centre for Cancer Genetic Epidemiology, Department of Public Health and Primary Care, University of Cambridge, Cambridge CB1 8RN, United Kingdom; Centre for Cancer Genetic Epidemiology, Department of Public Health and Primary Care, University of Cambridge, Cambridge CB1 8RN, United Kingdom; Centre for Cancer Genetic Epidemiology, Department of Public Health and Primary Care, University of Cambridge, Cambridge CB1 8RN, United Kingdom; Centre for Cancer Genetic Epidemiology, Department of Public Health and Primary Care, University of Cambridge, Cambridge CB1 8RN, United Kingdom; Centre for Cancer Genetic Epidemiology, Department of Oncology, University of Cambridge, Cambridge CB1 8RN, United Kingdom; Centre for Cancer Genetic Epidemiology, Department of Public Health and Primary Care, University of Cambridge, Cambridge CB1 8RN, United Kingdom; Genomics Center, Centre Hospitalier Universitaire de Québec—Université Laval Research Center, Université Laval, Québec City, QC G1V 4G2, Canada; Centre for Cancer Genetic Epidemiology, Department of Public Health and Primary Care, University of Cambridge, Cambridge CB1 8RN, United Kingdom; Centre for Cancer Genetic Epidemiology, Department of Oncology, University of Cambridge, Cambridge CB1 8RN, United Kingdom; Centre for Cancer Genetic Epidemiology, Department of Public Health and Primary Care, University of Cambridge, Cambridge CB1 8RN, United Kingdom; Centre for Cancer Genetic Epidemiology, Department of Public Health and Primary Care, University of Cambridge, Cambridge CB1 8RN, United Kingdom

## Abstract

**Background:**

The Breast and Ovarian Analysis of Disease Incidence Algorithm (BOADICEA) model predicts breast cancer risk using cancer family history, epidemiological, and genetic data. We evaluated its validity in a large prospective cohort.

**Methods:**

We assessed model calibration, discrimination and risk classification ability in 217 885 women (6838 incident breast cancers) aged 40-70 years of self-reported White ethnicity with no previous cancer from the UK Biobank. Age-specific risk classification was assessed using relative risk thresholds equivalent to the absolute lifetime risk categories of less than 17%, 17%-30%, and 30% or more, recommended by the National Institute for Health and Care Excellence guidelines. We predicted 10-year risks using BOADICEA v.6 considering cancer family history, questionnaire-based risk factors, a 313–single nucleotide polymorphisms polygenic score, and pathogenic variants. Mammographic density data were not available.

**Results:**

The polygenic risk score was the most discriminative risk factor (area under the curve [AUC] = 0.65). Discrimination was highest when considering all risk factors (AUC = 0.66). The model was well calibrated overall (expected-to-observed ratio = 0.99, 95% confidence interval [CI] = 0.97 to 1.02; calibration slope = 0.99, 95% CI = 0.99 to 1.00), and in deciles of predicted risks. Discrimination was similar in women aged younger and older than 50 years. There was some underprediction in women aged younger than 50 years (expected-to-observed ratio = 0.89, 95% CI = 0.84 to 0.94; calibration slope = 0.96, 95% CI = 0.94 to 0.97), which was explained by the higher breast cancer incidence in UK Biobank than the UK population incidence in this age group. The model classified 87.2%, 11.4%, and 1.4% of women in relative risk categories less than 1.6, 1.6-3.1, and at least 3.1, identifying 25.6% of incident breast cancer patients in category relative risk of at least 1.6.

**Conclusion:**

BOADICEA, implemented in CanRisk (www.canrisk.org), provides valid 10-year breast cancer risk, which can facilitate risk-stratified screening and personalized breast cancer risk management.

## Introduction

Female breast cancer is common, with approximately 2.3 million incident patients and 685 000 deaths per year worldwide.^1^ Breast cancer screening and risk-reduction options, such as risk-reducing surgery and risk-reducing medication, are available but are associated with overdiagnosis and overtreatment and may be associated with adverse side effects.^2^^,^^3^ Breast cancer risk prediction models can facilitate risk stratification thus enabling targeting screening and prevention strategies to those most likely to benefit, ultimately improving the benefit-to-harm ratio.^2^

A wide range of epidemiological factors are known to be associated with breast cancer risk including age; cancer family history;^4^ questionnaire-based risk factors including reproductive, hormonal and lifestyle factors;^5^^,^^6^ and mammographic density.^5^ Rare pathogenic genetic variants in *BRCA1*, *BRCA2*, *ATM*, *CHEK2*, *RAD51C*, *RAD51D*, *BARD1*, and *PALB2* are also associated with high or moderate risks of developing breast cancer.^7-11^ Furthermore, the combined effects of common, low-risk single nucleotide polymorphisms identified through genome-wide association studies, summarized in polygenic risk scores, have been shown to lead to clinically meaningful levels of breast cancer risk stratification.^12^ The latest version (v.6) of the multifactorial Breast and Ovarian Analysis of Disease Incidence Algorithm (BOADICEA)^13^^,^^14^ incorporates all the above established risk factors to predict the future risk of developing breast cancer (Table S1). It is implemented in the CanRisk tool (www.canrisk.org) that can be used by health-care professionals to obtain cancer risks easily.^13^^,^^15^

The performance of BOADICEA v.6 was previously evaluated in a Swedish screening cohort of 66 415 women and was shown to discriminate well and provide well-calibrated risks in different risk categories when predicting 5-year breast cancer risks.^16^ Here, we assessed BOADICEA v.6 performance in predicting longer term, 10-year risks in a much larger volunteer prospective cohort of 217 885 women from the UK Biobank, which included all information on all the risk predictors considered by the BOADICEA model, except mammographic density.

## Methods

### Study participants

**Figure 1.**
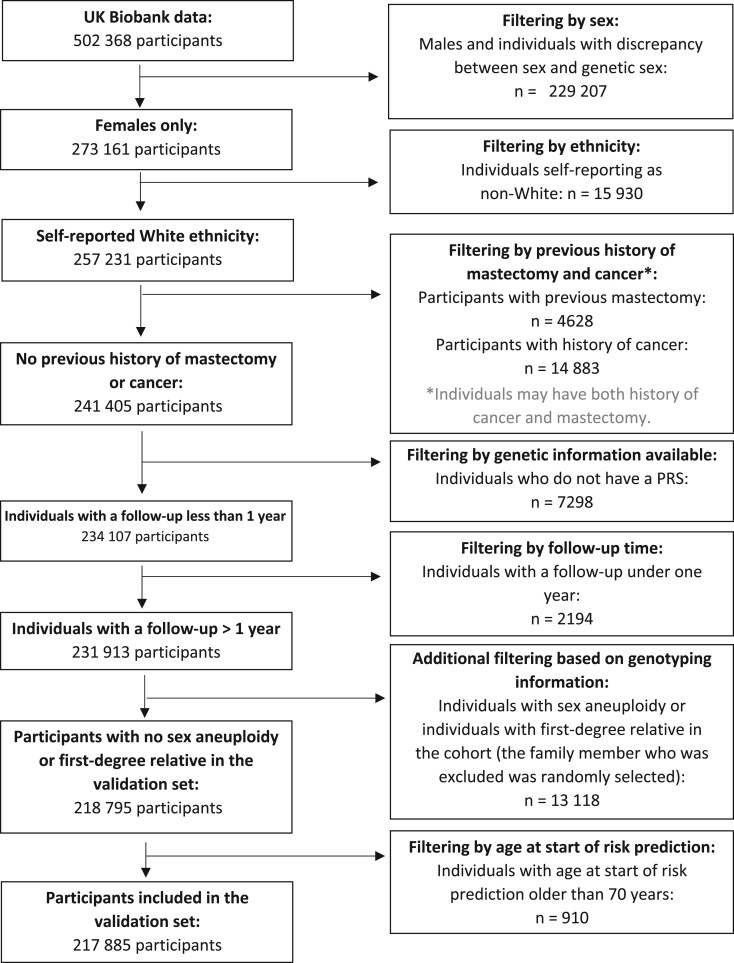
Flowchart summarizing selection of participants in the UK Biobank. PRS = polygenic risk scores.

The UK Biobank recruited 502 413 participants across the United Kingdom between 2006 and 2010^17^ (Supplementary Materials). We selected women aged younger than 70 years at recruitment, without previous cancer diagnosis (any except nonmelanoma skin cancer) or bilateral prophylactic mastectomy history, with follow-up of more than 1 year and with genotyping information available. For the main analysis, we focused on self-reported White women. Additionally, we conducted a separate sensitivity analysis on non-White women. Details of participant selection is summarized in Figure 1. Questionnaire-based risk factors including age at menarche, age at first live birth, parity, age at menopause, age at baseline, alcohol intake, and body mass index were collected at recruitment. Summary data on family history of breast and prostate cancer among first-degree relatives were available in UK Biobank and were used to construct the pedigree format family history required in the BOADICEA algorithm (details in Supplementary Material). All study participants provided informed consent and UK Biobank has approval from the North West Multi-centre research ethics committee as a research tissue bank.

### Genetic data

The UK Biobank participant samples were genotyped using the Affymetrix UK BiLEVE Axiom array and Affymetrix UK Biobank Axiom array. Imputation was performed using SHAPEIT3 and IMPUTE3^18^ to the combined 1000 Genomes Project v.3 and UK10K reference panels. The imputed data were used to derive the 313–single nucleotide polymorphisms polygenic risk score,^12^ which was standardized using a mean of -0.424 and standard deviation of 0.603 as reported by Mavaddat et al.^12^ The whole exome sequencing data were used to determine pathogenic genetic variant carrier status in the *BRCA1*, *BRCA2*, *PALB2*, *ATM*, *CHEK2*, *RAD51C*, *RAD51D*, and *BARD1*. Frameshift, nonsense, canonical splice site variants and large genomic deletions were considered as pathogenic genetic variants except for those in the last exons or the last 50 bp of the penultimate exons.

### Risk prediction

Eligible participants were followed from the age at recruitment until the first of the following events: the date of cancer (any), mastectomy, death, last linkage date, the end of the 10-year risk prediction period, or age 80 years. We predicted 10-year breast cancer risks using BOADICEA v.6^13^^,^^14^ with age- and calendar period–specific UK cancer incidences starting from the age at recruitment plus 1 year to exclude any potential prevalent breast cancers. For unaffected women with shorter follow-up time (<10 years), the risks were predicted to the age at censoring. BOADICEA allows for missing data by using the average population effects over the missing risk factor categories.^13^

### Statistical analysis

We assessed overall model calibration by calculating the ratio of expected to observed (E/O) number of breast cancer patients. The 95% confidence intervals (CIs) for E/O ratios were calculated using the normal approximation to the Poisson distribution:^19^


$$95\%\;CI\;\left(\frac{E}{O}\right)=\frac{E}{O}*\exp\left(\pm 1.96*\sqrt{\frac{1}{O}}\right).$$

Calibration slope was used to assess the agreement between the observed and predicted breast cancer risk for each individual, which was estimated by fitting a logistic regression where the outcome is the observed breast cancer status and the predictor is the log-odds of the predicted risk. To assess the model calibration across different risk categories, we contrasted the mean predicted risk against the observed risk by grouping women on the basis of the deciles of predicted risks, using calibration plots.

The discriminative ability was assessed by the area under the curve (AUC) and the Harrell C statistic.^20^ We assessed the model performance by using different combinations of the risk factors and separately in women aged younger and older than 50 years and separately in identified pathogenic genetic variant carriers.

We assessed the model’s risk classification ability by calculating the proportions of all individuals and incident breast cancer patients in different risk groups. Age-independent and age-dependent absolute risk thresholds were used in defining risk groups. To use age-dependent risk thresholds, we converted the predicted 10-year risks into relative risks (RRs), relative to the population age-specific average absolute risk.^21^ To categorize women into the risk categories, we used relative risk thresholds of less than 1.6, 1.6-3.1, and at least 3.1, which are equivalent to absolute lifetime risk categories of less than 17%, 17%-30%, and 30% or more, recommended by the National Institute for Health and Care Excellence (NICE) guidelines.^3^ For the age-independent risk categorization, we considered women with 10-year absolute breast cancer risks of less than 3%, 3%-8%, and 8% or more to be in the near-population, moderate, and high-risk categories, respectively, as suggested by the NICE guidelines.^3^ We also considered the alternative 10-year absolute breast cancer risk threshold of less than 5.8%, 5.8%-11%, and at least 11%, corresponding to the equivalent 10-year risk thresholds for a woman at the median age of 58 years observed in the UK Biobank and using the relative risk thresholds. Analyses were conducted in R (version 4.1.0)^22^ and Stata (version 17.1).^23^

## Results

The analysis included 217 885 White women aged 40-70 years, among whom 6838 women developed breast cancer within 10 years of follow-up. Cohort characteristics are shown in Table 1. On average, the incidences in UK Biobank were 58.19% higher than the population incidences in women aged younger than 50 years and 2.65% higher in women aged 50 years and older (Figure S1).

**Table 1.** Characteristics of participants of white ethnicity in the UK Biobank validation cohort^a^

		Healthy participants	Patients
Variables	Values	No. (%)	No. (%)
Participants, No.		211 047	6838
Genetic risk factors, carrier			
*BRCA1*		93 (0.0)	14 (0.2)
*BRCA2*		412 (0.2)	64 (1.0)
*PALB2*		282 (0.1)	28 (0.4)
*ATM*		451 (0.2)	33 (0.5)
*CHEK2*		933 (0.5)	62 (1.0)
*RAD51C*		60 (0.0)	2 (0.0)
*RAD51D*		87 (0.0)	2 (0.0)
*BARD1*		110 (0.1)	7 (0.1)
Standardized polygenic risk score, mean (SD)		0.020 (1.0)	0.482 (1.0)
Epidemiological risk factors			
Age at start of risk prediction, y	40-44	15 991 (7.6)	402 (5.9)
	45-49	27 528 (13.0)	789 (11.5)
	50-54	33 079 (15.7)	922 (13.5)
	55-59	38 304 (18.1)	1233 (18.0)
	60-64	51 088 (24.2)	1857 (27.2)
	65-69	45 057 (21.3)	1635 (23.9)
Age at start of risk prediction, mean (SD)		57.3 (7.9)	58.2 (7.7)
Follow-up, mean (SD)		9.7 (1.3)	5.4 (2.8)
Age at menarche, y	≤10	9394 (4.6)	329 (4.9)
	11	31 852 (15.5)	1034 (15.5)
	12	38 757 (18.9)	1340 (20.1)
	13	50 352 (24.5)	1654 (24.8)
	14	40 678 (19.8)	1265 (19.0)
	15	22 545 (11.0)	653 (9.8)
	≥16	10 699 (5.2)	362 (5.4)
	Missing	5914	172
Age at menopause, y	≤39	4706 (4.0)	139 (3.5)
	40-44	10 568 (8.9)	298 (7.5)
	45-49	28 003 (23.5)	837 (21.0)
	50-54	57 652 (48.4)	1963 (49.2)
	55-70	18 086 (15.2)	756 (18.9)
	Missing	92 032	2845
Parity	0	39 581 (18.8)	1344 (19.7)
	1	27 959 (13.3)	923 (13.5)
	2	93 809 (44.5)	3040 (44.5)
	≥3	49 567 (23.5)	1526 (22.3)
	Missing	131	5
Age at first live birth among parous women, y	≤19	13 877 (9.7)	421 (9.2)
	20-24	49 526 (34.6)	1541 (33.8)
	25-29	54 233 (37.9)	1772 (38.8)
	≥30	25 595 (17.9)	828 (18.1)
	Missing	28 103	927
Oral contraceptive use	Current	8402 (4.0)	285 (4.2)
	Former	165 639 (78.6)	5292 (77.5)
	Never	36 591 (17.4)	1250 (18.3)
Menopausal hormone therapy use	Current C type	25 093 (21.1)	990 (24.8)
	Current E type	296 (0.2)	14 (0.4)
	Former	30 247 (25.4)	1022 (25.6)
	Never	64 036 (53.8)	2006 (50.2)
Body mass index, kg/m^2^	[0,18.5)	1581 (0.8)	37 (0.5)
	[18.5,25)	83 639 (39.8)	2475 (36.3)
	[25,30)	76 972 (36.6)	2607 (38.2)
	[30,80]	48 216 (22.9)	1697 (24.9)
	Missing	639	22
Body mass index, mean (SD)		27.0 (5.1)	27.4 (5.1)
Height, cm	[0,153)	10 498 (5.0)	290 (4.3)
	[153,160)	54 439 (25.8)	1644 (24.1)
	[160,166)	79 118 (37.6)	2523 (37.0)
	[166,173)	54 398 (25.8)	1914 (28.1)
	[173,200]	12 167 (5.8)	452 (6.6)
	Missing	427	15
Height, mean (SD)		162.6 (6.2)	163.1 (6.3)
Alcohol, g/day	[0,5)	51 277 (30.7)	1590 (29.1)
	[5,15)	66 951 (40.1)	2121 (38.8)
	[15,25)	30 000 (18.0)	1096 (20.0)
	[25,35)	11 274 (6.8)	369 (6.7)
	[35,45)	4107 (2.5)	157 (2.9)
	[45,100]	3184 (1.9)	134 (2.4)
	Missing	44 106	1366

a The main analysis was restricted to women of self-reported White ethnicity.

### Model discrimination and calibration

**Figure 2.**
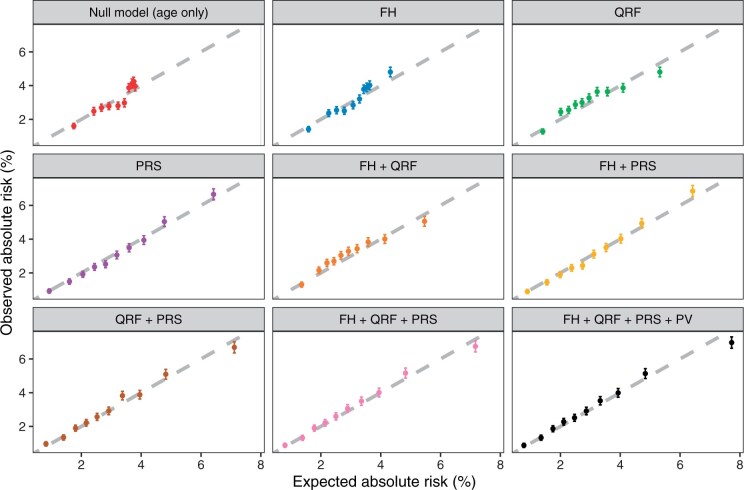
Calibration of 10-year predicted breast cancer risks under different risk factor combinations in Breast and Ovarian Analysis of Disease Incidence Algorithm. FH = family history; QRF = questionnaire-based risk factors; PRS = polygenic risk scores; PV = pathogenic variants.

Compared with the model that considered age only, the addition of each risk factor provided a wider distribution of predicted risks and increased the discriminatory ability (Figure S2, Table 2). Among the individual risk factors, the polygenic risk score provided the largest increase in discriminative ability (AUC = 0.65, 95% CI = 0.65 to 0.66), followed by family history (AUC = 0.59, 95% CI = 0.58 to 0.60) and questionnaire-based risk factors (AUC = 0.58, 95% CI = 0.58 to 0.59). When polygenic risk score, family history, questionnaire-based risk factors, and pathogenic genetic variants were jointly considered, the discriminative ability was highest (AUC = 0.66, 95% CI = 0.66 to 0.67), and the model provided the widest range of risks (Table 2, Figure S2). The full model was well calibrated overall (calibration slope = 0.99, 95% CI = 0.99 to 1.00; E/O = 0.99, 95% CI = 0.97 to 1.02) and in deciles of predicted risks (Table 2, Figure 2).

**Table 2.** Calibration and discrimination statistics for the 10-year predicted Breast and Ovarian Analysis of Disease Incidence Algorithm risks

Model	Calibration slope (95% CI)	Expected-to-observed ratio (95% CI)	Area under the curve (95% CI)	Harrell C statistic (95% CI)
White women				
Null model, age only	1.00 (0.99 to 1.01)	0.99 (0.97 to 1.01)	0.56 (0.56 to 0.57)	0.56 (0.56 to 0.57)
FH	0.99 (0.98 to 1.00)	0.97 (0.95 to 0.99)	0.59 (0.58 to 0.60)	0.58 (0.57 to 0.58)
QRF	0.99 (0.98 to 0.99)	0.96 (0.94 to 0.98)	0.58 (0.58 to 0.59)	0.57 (0.56 to 0.58)
PRS	1.01 (1.00 to 1.02)	1.02 (0.99 to 1.04)	0.65 (0.65 to 0.66)	0.64 (0.63 to 0.65)
FH + QRF	0.98 (0.98 to 0.99)	0.95 (0.93 to 0.98)	0.59 (0.59 to 0.60)	0.58 (0.57 to 0.59)
FH + PRS	1.00 (0.99 to 1.01)	1.00 (0.98 to 1.02)	0.66 (0.65 to 0.66)	0.64 (0.64 to 0.65)
QRF + PRS	0.99 (0.98 to 1.00)	0.98 (0.96 to 1.01)	0.65 (0.65 to 0.66)	0.64 (0.64 to 0.65)
FH + QRF + PRS	0.99 (0.98 to 1.00)	0.98 (0.96 to 1.00)	0.66 (0.65 to 0.66)	0.65 (0.64 to 0.66)
FH + PRS + QRF + PV	0.99 (0.99 to 1.00)	0.99 (0.97 to 1.02)	0.66 (0.66 to 0.67)	0.65 (0.65 to 0.66)
White women aged younger than 50 years				
PRS	0.96 (0.95 to 0.98)	0.90 (0.85 to 0.95)	0.64 (0.62 to 0.65)	0.63 (0.62 to 0.65)
FH + QRF + PRS	0.96 (0.94 to 0.98)	0.88 (0.83 to 0.93)	0.65 (0.63 to 0.66)	0.64 (0.63 to 0.66)
FH + PRS + QRF + PV	0.96 (0.94 to 0.97)	0.89 (0.84 to 0.94)	0.66 (0.64 to 0.67)	0.65 (0.63 to 0.67)
White women aged 50 years and older				
PRS	1.02 (1.01 to 1.03)	1.05 (1.02 to 1.07)	0.65 (0.64 to 0.66)	0.64 (0.63 to 0.64)
FH + QRF + PRS	1.00 (0.99 to 1.01)	1.01 (0.98 to 1.04)	0.66 (0.65 to 0.66)	0.64 (0.64 to 0.65)
FH + PRS + QRF + PV	1.00 (0.99 to 1.01)	1.01 (0.99 to 1.04)	0.66 (0.65 to 0.67)	0.65 (0.64 to 0.66)
White pathogenic variant carriers				
PV	1.09 (1.01 to 1.17)	1.27 (1.11 to 1.45)	0.63 (0.59 to 0.67)	0.63 (0.59 to 0.66)
FH + QRF + PRS + PV	1.08 (1.00 to 1.16)	1.31 (1.14 to 1.50)	0.66 (0.62 to 0.70)	0.65 (0.61 to 0.68)
Non-White women				
FH + QRF + PRS + PV	1.64 (1.57 to 1.69)	4.02 (3.61 to 4.48)	0.54 (0.51 to 0.58)	0.55 (0.52 to 0.58)

Abbreviations: FH = family history; QRF = questionnaire-based risk factors; PRS = polygenic risk scores; PV = pathogenic variants.

### Model performance by age groups

**Figure 3.**
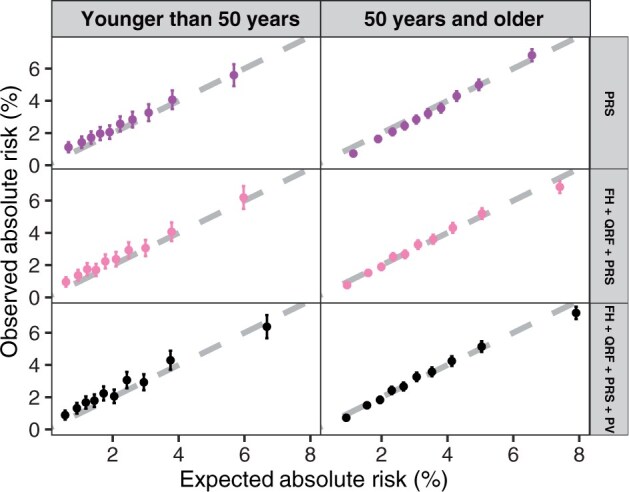
Calibration of 10-year predicted breast cancer risks under different risk factor combinations by age groups. FH = family history; QRF = questionnaire-based risk factors; PRS = polygenic risk scores; PV = pathogenic variants.

Among the eligible women, there were 44 710 women aged younger than 50 years at recruitment (1191 incident breast cancers) and 173 175 aged 50 years or older (5647 incident breast cancers) (Table S2). The discrimination was at its highest in the full model in women aged younger and older than 50 years (AUC = 0.66; Table 2). The model was well calibrated in women aged older than 50 years (overall calibration slope = 1.00, 95% CI = 0.99 to 1.01; overall E/O = 1.01, 95% CI = 0.99 to 1.04; Table 2, Figure 3), but there was an underprediction of risk among women aged younger than 50 years (calibration slope = 0.96, 95% CI = 0.94 to 0.97; E/O = 0.89, 95% CI = 0.84 to 0.94; Table 2, Figure 3).

### Model performance in pathogenic genetic variant carriers

**Figure 4.**
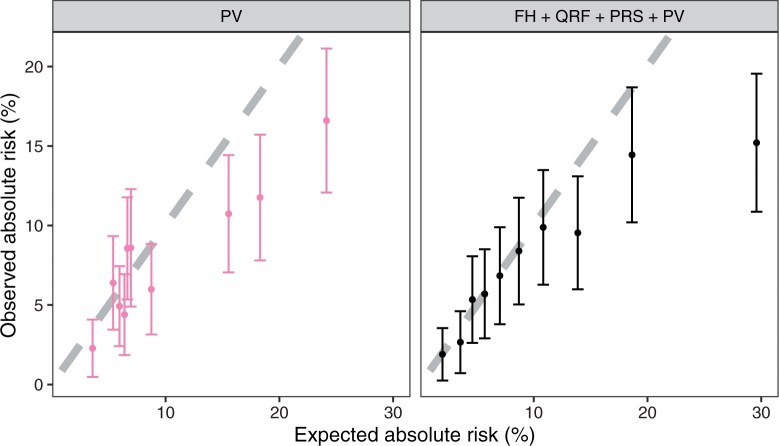
Calibration of 10-year predicted breast cancer risks in pathogenic variant carriers. FH = family history; QRF = questionnaire-based risk factors; PRS = polygenic risk scores; PV = pathogenic variants.

There were 2627 women (210 incident breast cancers) who carried a rare pathogenic genetic variant in 1 of the 8 breast cancer susceptibility genes. Among those, there were 107 *BRCA1*, 476 *BRCA2*, and 995 *CHEK2* PV carriers. The model considering pathogenic genetic variants alone overpredicted breast cancer risks in most deciles (Figure 4). The addition of family history, questionnaire-based risk factors, and polygenic risk score provided a wider range of risks and improved the model calibration across deciles of predicted risks especially for the lower- and middle-risk deciles. But there was an overprediction of risk, particularly for the top risk decile (Figure 4). The model discriminative ability was also improved by the inclusion of family history, questionnaire-based risk factors, and polygenic risk score in the model (AUC = 0.66, 95% CI = 0.62 to 0.70; Table 2).

### Model performance in non-White women

Although BOADICEA v.6 was parameterized using data on White women, we have assessed the model performance in all non-White UK Biobank participants combined. There were 14 243 non-White women including 335 incident breast cancer patients over 10 years of follow-up. Detailed characteristics are shown in Table S4. The full model overpredicted breast cancer risk with a calibration slope of 1.63 (95% CI = 1.57 to 1.69) and demonstrated poor discrimination with an AUC of 0.54 (95% CI = 0.51 to 0.58; Table 2). Given the need for the model to be specifically adapted to non-White ethnic groups, this sample was not considered in downstream analyses.

### Risk classification

The age-dependent and age-independent risk thresholds used for defining the 3 risk groups are shown in Figure S3. The model considering only epidemiological risk factors including family history and questionnaire-based risk factors identified 4.4% of women with relative risks of at least 1.6, 45.0% of women with 10-year breast cancer risk of at least 3%, and 2.8% of women with 10-year breast cancer risk of at least 5.8% (Table 3). When genetic risk factors were added, the full model classified 87.2%, 11.4%, and 1.4% women in the relative risk less than 1.6, 1.6-3.1, and at least 3.1 categories; 56.0%, 41.2%, and 2.8% women in 10-year risk less than 3%, 3%-8%, and at least 8%; and 91.1%, 8.1%, and 0.8% women in 10-year risk less than 5.8%, 5.8%-11%, and at least 11%, respectively (Table 3). When considering women at the top 10%, 30%, and 50% of the predicted risks as at risk, the full model identified 22%, 51%, and 72% of incident breast cancer patients (Table S3).

**Table 3.** Percentage of participants and breast cancer patients in different relative and absolute risk categories

Risk categories	Model: FH^a^ + QRF		Model: FH^a^ + QRF + PRS		Model: FH^a^ + QRF + PRS + PV	
	Population	Breast cancer patients	Population	Breast cancer patients	Population	Breast cancer patients
Relative risk thresholds						
All women						
<1.6	95.6	92.8	87.1	75.3	87.2	74.4
1.6 ≤ RR < 3.1	4.4	7.1	11.8	22.0	11.4	21.2
≥3.1	0.0	0.1	1.0	2.7	1.4	4.4
Women aged younger than 50 years						
<1.6	96.1	93.2	84.4	68.0	84.8	67.6
1.6 ≤ RR < 3.1	3.8	6.7	13.8	27.5	12.9	25.4
≥3.1	0.1	0.1	1.9	4.5	2.3	7.0
Women aged 50 years and older						
<1.6	95.4	92.7	87.9	76.8	87.9	75.8
1.6 ≤ RR < 3.1	4.5	7.2	11.3	20.8	11.0	20.3
≥3.1	0.0	0.1	0.8	2.4	1.2	3.9
Absolute 10-year breast cancer risk thresholds as recommended by National Institute for Health and Care Excellence guidelines						
All women						
<3%	55.0	45.6	55.0	35.4	56.0	35.5
3% ≤ risk < 8%	44.8	53.8	42.6	58.3	41.2	56.6
≥8%	0.2	0.6	2.4	6.3	2.8	7.9
Women aged younger than 50 years						
<3%	88.7	82.6	74.6	55.8	75.4	55.6
3% ≤ risk < 8%	11.3	17.4	24.4	41.6	23.1	39.2
≥8%	0.0	0.0	1.0	2.7	1.5	5.2
Women aged 50 years and older						
<3%	46.3	37.8	50.0	31.2	51.0	31.3
3% ≤ risk < 8%	53.5	61.5	47.3	61.8	45.8	60.2
≥8%	0.3	0.7	2.8	7.0	3.1	8.5
Alternative absolute 10-year breast cancer risk thresholds						
All women						
<5.8%	97.2	95.0	91.2	81.7	91.1	80.2
5.8% ≤ risk < 11%	2.8	4.9	8.3	16.9	8.1	16.8
≥11%	0.0	0.1	0.4	1.4	0.8	2.9
Women aged younger than 50 years						
<5.8%	99.7	99.4	95.8	90.1	95.5	88.1
5.8% ≤ risk < 11%	0.3	0.6	4.1	9.4	3.9	9.4
≥11%	0.0	0.0	0.2	0.5	0.6	2.5
Women aged 50 years and older						
<5.8%	96.6	94.0	90.1	79.9	89.9	78.6
5.8% ≤ risk < 11%	3.4	5.9	9.4	18.5	9.2	18.4
≥11%	0.0	0.1	0.5	1.5	0.9	3.0

Abbreviations: FH = family history; QRF = questionnaire-based risk factors; PRS = polygenic risk scores; PV = pathogenic variants; RR = relative risk.

a As collected in UK Biobank: summary cancer family history in first-degree relatives.

Using age-dependent absolute risk thresholds, the full model classified 12.9% and 2.3% of women aged younger than 50 years in the categories of relative risk between 1.6 and 3.1 and relative risk of at least 3.1, identifying 25.4% and 7.0% of incident breast cancer patients occurring in the 10-year period (Table 3). For women aged 50 years or older, 11.0% and 1.2% of women were classified in the categories of relative risk between 1.6 and 3.1 and relative risks of at least 3.1, identifying 20.3% and 3.9% of incident breast cancer patients, respectively (Table 3). Using the NICE-recommended and the alternative age-independent 10-year risk thresholds, there was a larger proportion of women classified as at higher risk among those in the age 50 years and older group than aged younger than 50 years, with 45.8% and 10.1% of women aged 50 years or older classified in the risk of at least 3% and risk of at least 5.8% categories, respectively, compared with 24.6% and 4.5% of women in the younger group, respectively (Table 3).

Among pathogenic genetic variant carriers, the model considering pathogenic genetic variants only did not classify any women in the near-population risk group (RR < 1.6). The full model classified 26.0%, 32.0%, and 41.9% of carriers in relative risk less than 1.6, relative risk between 1.6 and 3.1, and relative risk of at least  3.1 categories, identifying 61.9% of incident breast cancer patients in the high-risk group (Table 4). The NICE-recommended age-independent thresholds classified 41.2% and 51.4% of carriers in the categories of risk between 3% and 8%, and risk over 8%, identifying 27.1% and 70.5% of breast cancer patients, respectively (Table 4). Using the alternative age-independent 10-year risk thresholds, the full model classified 31.1% and 36.3% of carriers in the categories of risk between 5.8% and 11% and risk over 11%, identifying 30.0% and 53.3% of incident breast cancer patients, respectively.

**Table 4.** Percentage of pathogenic variant carriers and breast cancer patients with pathogenic variants in different relative and absolute risk categories

	Model: PV		Model: FH^a^ + QRF + PRS + PV	
Risk categories	Population	Breast cancer patients	Population	Breast cancer patients
Relative risk thresholds				
<1.6	0.0	0.0	26.0	13.3
1.6 ≤ RR < 3.1	66.0	49.5	32.0	24.8
≥3.1	34.0	50.5	41.9	61.9
Absolute 10-year breast cancer risk thresholds as recommended by National Institute for Health and Care Excellence guidelines				
<3%	0.0	0.0	7.3	2.4
3% ≤ risk < 8%	66.0	49.5	41.2	27.1
≥8%	34.0	50.5	51.4	70.5
Alternative absolute 10-year breast cancer risk thresholds				
<5.8%	16.3	11.9	32.7	16.7
5.8% ≤ risk < 11%	49.7	37.6	31.1	30.0
≥11%	34.0	50.5	36.3	53.3

Abbreviations: FH = family history; QRF = questionnaire-based risk factors; PRS = polygenic risk scores; PV = pathogenic variants.

a As collected in UK Biobank: summary cancer family history in first degree relatives.

## Discussion

This study evaluated the performance of the latest version of the BOADICEA model (version 6)^13^^,^^14^ in predicting 10-year breast cancer risks using the joint effects of family history, questionnaire-based risk factors, polygenic risk scores, and rare pathogenic genetic variants in the UK Biobank prospective cohort. This is the largest validation study to date with more than 200 000 women and 6838 incident breast cancer patients. The results show that BOADICEA is well calibrated overall and among women aged 50 years and older. There was some underprediction of risks for women aged younger than 50 years as measured by the calibration slope, but the predicted risks by deciles were in line with the observed risks among this group of younger women.

BOADICEA v.5 was previously assessed in 4 smaller population-based studies and showed good calibration consistently, with C statistics of 0.65-0.70 for predicting 5-year,^24^^,^^25^ 0.62 for 10-year,^26^ and 0.59 for 15-year risks.^27^ Only subsets of risk factors were considered in these studies including family history, questionnaire-based risk factors, and/or polygenic risk scores.^24-26^ BOADICEA v.6 was assessed in only 1 previous population-based study^16^ in predicting 5-year breast cancer risks in Swedish women where all risk factors including family history, questionnaire-based risk factors, polygenic risk scores, pathogenic genetic variants, and mammographic density were considered. The study showed BOADICEA to be well calibrated and to discriminate with an AUC of 0.70.

Here for the first time, we assessed the 10-year risk prediction of the latest BOADICEA v.6 in a much larger cohort. The results show that the addition of family history, questionnaire-based risk factors, and polygenic risk scores to age improves the model discriminative ability with the polygenic risk score being the most discriminative risk factor, which is consistent with previous studies.^16^^,^^24^^,^^26^ The addition of polygenic risk scores to family history and questionnaire-based risk factors identifies an extra approximately 8% of women (22 358 women) as being at moderate or high risk (using the relative risk thresholds), which captures an extra 17.5% of incident breast cancers (1317 patients) occurring during the risk prediction period. For the full BOADICEA model, as implemented here, there was some overprediction of breast cancer risk for women in the highest risk decile (which was restricted to those aged older than 50 years). If this is the case, it may potentially lead to more frequent screening or risk-reducing options for women who are classified at high risk in this age group. However, it should be noted that the predicted risk was very close to the observed risk for women in the highest-risk decile (Figures 2 and 3).

The Gail and the Tyrer–Cuzick models have previously been validated in postmenopausal women of White British ancestry from the UK Biobank.^28^ These models also showed improved performance with the inclusion of a breast cancer polygenic risk score: Tyrer–Cuzick C index of 0.57 (95% CI = 0.55 to 0.58), increasing to 0.67 (95% CI = 0.66 to 0.69) with polygenic risk score; Gail C index of 0.54 (95% CI = 0.52 to 0.56), increasing to 0.67 (95% CI = 0.65 to 0.68) with polygenic risk score. These findings are consistent with the present study. However, the reported AUCs may have been overestimated because the models including the polygenic risk score were developed using a cross-validation approach within the UK Biobank datasets. Therefore, the reported AUC values do not necessarily represent an independent validation. Despite model recalibration, within UK Biobank, the models also showed overall miscalibration with calibration slopes 1.10 to 1.11 for the Tyrer–Cuzick and Gail models, respectively.^28^ These compare with an estimated calibration slope of 0.99 in the present study. BOADICEA considers a more comprehensive set of risk factors, including pathogenic genetic variants in all established breast cancer susceptibility genes, and unlike the published validation, which focused solely on postmenopausal women,^28^ the present analysis provides evidence that that BOADICEA performs well in predicting risks for women across the age range of 40-69 years.

When the model performance was assessed by age group, BOADICEA showed similarly good discriminative ability in women aged younger or older than 50 years (Table 2). There was an underprediction in women aged younger than 50 years as measured by the calibration slope and ratio of expected to observed. However, this is most likely because of the higher incidence observed in the UK Biobank in women aged younger than 50 years compared with the general population incidences, which are used in BOADICEA (Figure S1). Moreover, the predicted risks by deciles were in line with the observed risks among this group of younger women.

Among pathogenic genetic variant carriers, the inclusion of family history, questionnaire-based risk factors, and polygenic risk score in addition to pathogenic genetic variant status improved the model discriminative ability from an AUC of 0.63 (95% CI = 0.59 to 0.67) to 0.66 (95% CI = 0.62 to 0.70) and improved the model calibration substantially especially for the low- and moderate-risk women. There was some overprediction in the highest risk decile among whom 191 of 262 pathogenic genetic variant carriers in the decile were 50 years or older: 76 carried *BRCA1* and 125 *BRCA2* pathogenic genetic variants. A recent study of a larger sample of *BRCA1* and *BRCA2* pathogenic genetic variant carriers identified through clinical genetics centers suggested a similar pattern for older high-risk *BRCA2* pathogenic genetic variant carriers although the overprediction was not statistically significant.^29^ The observation in the present study could be a consequence of a much smaller sample size of pathogenic genetic variant carriers but also because granular family history of cancer is not available among relatives of UK Biobank participants. Specifically, information on unaffected family members is incomplete, which may lead to some overestimation of risk, when considering primarily information on affected relatives in the risk prediction. When only pathogenic genetic variant information was considered, none of the women were classified in the low-risk group using the age-dependent risk thresholds (Table 4). The addition of all other risk factors reclassified 26.04% of women into the relative risk less than 1.6 group. Among these women, 95.9% remained unaffected during the follow-up, suggesting that multifactorial risk assessment for pathogenic genetic variant carriers may help identify near-population risk women and may influence clinical management decisions.

As the incidence of breast cancer increases substantially with age, it was previously suggested that the use of age-independent absolute risk thresholds has the potential of missing high-risk younger women who would go on to develop breast cancer.^21^ This was demonstrated in the present analysis where using relative risk thresholds to identify women in the moderate- and high-risk categories identified 32.4% of incident breast cancer compared with 11.9% when using the equivalent absolute risk threshold of at least 5.8% among women aged younger than 50 years (Table 3).

When using the NICE-recommended 10-year risk threshold of at least 3%, the full model identified 44.4% of incident breast cancer among women aged younger than 50 years as moderate and high risk. As the 10-year NICE-recommended risk thresholds were originally calculated for women aged 40 years, as expected, a higher proportion of women were classified in the moderate or high-risk groups among women aged older than 50 years (48.9%) and identified 68.7% of the incident cancers in this group. Previous studies have also found that the use of fixed horizon risk (5-year risks) thresholds compared with predicted lifetime or remaining risks yields higher specificity in younger women, reducing the harms associated with unnecessary screening, risk-reducing surgery, or medication.^30^

This study has several limitations. First, although BOADICEA considers mammographic density, this is not available in the UK Biobank dataset. Previous studies found that mammographic density is one of the strongest risk factors for breast cancer.^31^ Second, self-reported family history in the UK Biobank was only available for breast and prostate cancer in the first-degree relatives. Summarized family history was reported with the number of affected siblings and age at diagnosis unknown. Family history beyond the first-degree relatives and family history of ovarian and pancreatic cancers, which are also included in BOADICEA, were not collected in the UK Biobank. Because BOADICEA uses pedigree-structured family history information in the risk prediction, a series of assumptions were used to construct the pedigrees such as only one sibling was assumed to be affected if cancer history of siblings was reported. The lack of granular family history information in the UK Biobank, combined with the fact that the majority of the women were aged older than 50 years, may explain the lower discriminative ability of the family history model compared with previous studies^16^ and the possible underestimation in high-risk women when considering family history and age only (Figure 2). It is expected that the prediction accuracy will improve if more granular, pedigree-based data are available. Third, 14 006 women were censored as unaffected within the 10-year risk prediction horizon (before reaching the end of the follow-up). For these women, the breast cancer risks were predicted to the censoring age, which might potentially lead to an overestimation in the AUC estimation. To adjust for this, we also estimated the Harrell C index that takes the survival time into consideration.^20^^,^^32^ The results were similar to the AUC estimates, and the conclusions were unaffected (Table 2). Fourth, the validation performed in this study and a previous published study^16^ was focused on women of White ethnicity, because of the small numbers of non-White ethnicity women available (a total of 14 243 eligible women with 335 incident breast cancer patients across all other ethnic groups combined; Table S4). Additionally, BOADICEA v.6 was developed using data on women of European ancestry and does not account for differences in cancer incidences and in the distributions of lifestyle and hormonal risk factors by ethnicity^33^ or variations in the performance of the polygenic risk score across different genetic ancestries.^12^^,^^34-37^ As expected, the BOADICEA v.6, without adjustments for ethnicity, had poor performance in non-White populations (Table 2). It is therefore critical to extend the model to non-European, non-White individuals, and further validation studies of BOADICEA in predicting the risks in these populations are required.

In conclusion, this is the largest validation study of the BOADICEA model to date. The results demonstrate that the latest comprehensive BOADICEA model is a clinically valid risk prediction tool with good overall calibration and discrimination across women between ages 40 and 70 years. It is implemented in the CanRisk webtool (www.canrisk.org),^15^ which can be used by health-care professionals in guiding personalized breast cancer risk management.

## Supplementary Material

djae335_Supplementary_Data

## Data Availability

Requests for UK Biobank should be made to the UK Biobank Access Management Team.
