# Supplementary material for: Evaluating the performance of the Breast and Ovarian Analysis of Disease Incidence Algorithm model in predicting 10-year breast cancer risks in UK Biobank
Source: J Natl Cancer Inst. 2024 Dec 12;117(5):948–58. doi: 10.1093/jnci/djae335 (PMC12058261; doi:10.1093/jnci/djae335)
Supplement: djae335_Supplementary_Data [file djae335_supplementary_data.pdf]

Supplementary material

Supplementary Methods

UK Biobank

Cancer diagnosis information and death were obtained through linkages with NHS Digital and Public Health England (for participants in England and Wales) and through the NHS Central Register (for participants in Scotland). This analysis was performed on the most recent update of patient information in the UK Biobank on the 1st of June 2022. Cancer types were encoded using ICD-10 (International Classification of Diseases 10) codes. When multiple first-degree family members were in the cohort, only one participant was kept (at random).

Pedigree construction

The pedigrees were constructed and taken into account for all women except for those reported to be adopted. For each participant, the UK Biobank collected data on the ages of parents, the number of biological brothers, the number of biological sisters and self-reported breast and prostate cancer status of the mother, father, and siblings (any) separately. If the proband has many siblings, it is unknown who developed cancer.

Based on this summarized family history information, we constructed family pedigrees for each individual under the following steps:

1. Determine Parents' Cancer Status and Age:
    - Record the mother's and father's cancer status (breast cancer for mother, prostate cancer for father).
    - If the parent had cancer and is deceased, use their age at death as the age of cancer diagnosis.
    - If the parent is alive, use their age at baseline as the last follow-up age.
2. Determine Siblings' Cancer Status and Age:
    - If there are any affected siblings:
        - Assume only one sibling is affected.
        - If it is breast cancer, check if the proband has any sisters:
            - If yes, assume the affected sibling is a sister.
            - If no, assume the affected sibling is a brother.
    - Record the age of cancer diagnosis or last follow-up age for siblings, assuming it matches the proband's age.

Supplementary table 1 – Breast cancer risk factors considered by BOADICEA.

Risk factors	Details	Availability in UK Biobank
Age		Available
Family and personal-proband history of cancer	Including breast, ovarian, pancreatic and prostate cancer	Family history of breast and prostate cancer available for first-degree relatives
Age information of unaffected family members		Available for parents
Year of birth	To capture information on birth cohort	Available
Reproductive and hormonal factors	Age at menarche, age at first live birth, parity, late menopause, the use of combined oral contraceptives and menopause hormone therapy	Available
Alcohol consumption	Daily alcohol intake in grams	Frequency of alcohol consumption was available in the UK Biobank. It was converted in UK units by assuming the standard strength (ABV) and volume of the drink, and further converted in grams of alcohol. It was assumed that 1 UK unit of alcohol corresponded to eight grammes of alcohol.
Body mass index		Available
Height		Available
Mammographic density		Not available
Ashkenazi Jewish origin		Not available
Common SNPs	Summarised through the 313-SNPs polygenic risk score (1)	Available
Rare pathogenic variants	*BRCA1, BRCA2, ATM, CHEK2, RAD51C, RAD51D, BARD1,* and *PALB2*	Available

Supplementary table 2 - Participant characteristics of the UK Biobank validation cohort by age group, among those of self-reported White ethnicity

		Women younger than 50				Women aged 50 and older			
		Controls		Cases		Controls		Cases	
Variables	Values	N	%	N	%	N	%	N	%
Participants	N	43,519	97.3	1,191	2.7	167,528	96.7	5,647	3.3
Genetic risk factors									
BRCA1	Carrier	32	0.1	5	0.4	61	0.0	9	0.2
BRCA2	Carrier	101	0.2	12	1.1	311	0.2	52	1.0
PALB2	Carrier	67	0.2	8	0.7	215	0.1	20	0.4
ATM	Carrier	102	0.3	10	0.9	349	0.2	23	0.4
CHEK2	Carrier	212	0.5	10	0.9	721	0.5	52	1.0
RAD51C	Carrier	20	0.0	0	0.0	40	0.0	2	0.0
RAD51D	Carrier	17	0.0	1	0.1	70	0.0	1	0.0
BARD1	Carrier	21	0.1	1	0.1	89	0.1	6	0.1
Standardised PRS	Mean	0.043	-	0.502	-	0.015	-	0.478	-
	SD	1.0	-	1.0	-	1.0	-	1.0	-
Epidemiological risk factors									

Age at start of risk prediction	[40,45)	15,991	36.7	402	33.8	-	-	-	-
	[45,50)	27,528	63.3	789	66.2	-	-	-	-
	[50,55)	-	-	-	-	33,079	19.7	922	16.3
	[55,60)	-	-	-	-	38,304	22.9	1,233	21.8
	[60,65)	-	-	-	-	51,088	30.5	1,857	32.9
	[65,70]	-	-	-	-	45,057	26.9	1,635	29.0
	Mean	45.5	-	45.6	-	60.3	-	60.9	-
	SD	2.4	-	2.4	-	5.6	-	5.4	-
Follow up	Mean	9.9	-	5.4	-	9.7	-	5.3	-
	SD	0.9	-	2.8	-	1.4	-	2.8	-
Age at menarche	[0,11)	1,729	4.1	56	4.8	7,665	4.7	273	5.0
	[11,12)	5,853	13.9	162	13.9	25,999	16.0	872	15.8
	[12,13)	7,927	18.8	230	19.8	30,830	18.9	1,110	20.2
	[13,14)	10,997	26.0	322	27.7	39,355	24.2	1,332	24.2
	[14,15)	8,553	20.2	216	18.6	32,125	19.7	1,049	19.1
	[15,16)	4,671	11.1	106	9.1	17,874	11.0	547	9.9
	[16,17]	2,323	5.5	61	5.2	8,376	5.1	301	5.5
	Missing	1,264	-	29	-	4,650	-	143	-
Age at menopause	[0,40)	462	20.4	10	23.3	4,244	3.6	129	3.3
	[40,45)	822	36.3	19	44.2	9,746	8.3	279	7.1

	[45,50)	981	43.3	14	32.6	27,022	23.1	823	20.8
	[50,55)	-	-	-	-	57,652	49.4	1,963	49.7
	[55,70]	-	-	-	-	18,086	15.5	756	19.1
	Missing	41,254	-	1,148	-	50,778	-	1,697	-
Parity	[0,1)	12,163	28.0	380	31.9	27,418	16.4	964	17.1
	[1,2)	6,991	16.1	191	16.0	20,968	12.5	732	13.0
	[2,3)	16,794	38.6	444	37.3	77,015	46.0	2,596	46.0
	[3,20]	7,550	17.4	176	14.8	42,017	25.1	1,350	23.9
	Missing	21	-	0	-	110	-	5	-
Age at first live birth among parous women	[0,20)	1,904	7.8	35	5.7	11,973	10.1	386	9.8
	[20,25)	5,969	24.5	133	21.5	43,557	36.6	1,408	35.7
	[25,30)	8,918	36.7	241	38.9	45,315	38.1	1,531	38.8
	[30,50]	7,527	31.0	210	33.9	18,068	15.2	618	15.7
	Missing	7,017	-	192	-	21,086	-	735	-
OC use	Current	6,235	14.4	221	18.6	2,167	1.3	64	1.1
	Former	33,363	76.8	867	72.8	132,276	79.1	4,425	78.5
	Never	3,837	8.8	103	8.6	32,754	19.6	1,147	20.4
MHT use	Current C-type	805	35.5	16	37.2	24,288	20.8	974	24.7
	Current E-type	17	0.8	0	0.0	279	0.2	14	0.4

	Former	94	4.2	1	2.3	30,153	25.8	1,021	25.8
	Never	1,382	61.0	26	60.5	62,654	53.7	1,980	50.1
BMI (kg/m2)	[0,18.5)	383	0.9	14	1.2	1,198	0.7	23	0.4
	[18.5,25)	20,655	47.6	576	48.4	62,984	37.7	1,899	33.7
	[25,30)	13,808	31.8	368	31.0	63,164	37.8	2,239	39.8
	[30,80]	8,554	19.7	231	19.4	39,662	23.7	1,466	26.1
	Missing	119	-	2	-	520	-	20	-
	Mean	26.3	-	26.2	-	27.2	-	27.6	-
	SD	5.3	-	5.0	-	5.1	-	5.1	-
Height (cm)	[0,153)	1,140	2.6	31	2.6	9,358	5.6	259	4.6
	[153,160)	8,095	18.6	209	17.6	46,344	27.7	1,435	25.5
	[160,166)	15,744	36.2	381	32.0	63,374	37.9	2,142	38.0
	[166,173)	14,176	32.6	434	36.5	40,222	24.1	1,480	26.3
	[173,200]	4,280	9.9	135	11.3	7,887	4.7	317	5.6
	Missing	84	-	1	-	343	-	14	-
	Mean	164.4	-	165.0	-	162.2	-	162.7	-
	SD	6.3	-	6.5	-	6.2	-	6.2	-
Alcohol (g/day)	[0,5)	9,220	26.8	245	25.5	42,057	31.7	1,345	29.8
	[5,15)	14,045	40.9	378	39.3	52,906	39.9	1,743	38.6

[15,25)	6,659	19.4	209	21.7	23,341	17.6	887	19.7
[25,35)	2,539	7.4	63	6.5	8,735	6.6	306	6.8
[35,45)	977	2.8	30	3.1	3,130	2.4	127	2.8
[45,100]	869	2.5	35	3.6	2,315	1.7	99	2.2
Missing	9,161	-	229	-	34,945	-	1,137	-

Supplementary table 3 - Performance measures using different positive classification thresholds as a percentage of top predicted risk under the model considering family history, questionnaire-risk factors, polygenic risk score and pathogenic variants

Top X% risk in cohort	5%	10%	20%	30%	40%	50%
FH + QRF + PRS + PV						
Proportion of incident BC patients with risks above the threshold (95% CI)	0.13<br>(0.12-0.14)	0.22<br>(0.21-0.23)	0.39<br>(0.37-0.40)	0.51<br>(0.50-0.52)	0.63<br>(0.61-0.64)	0.72<br>(0.71-0.73)
Proportion of healthy women with risks below the threshold (95% CI)	0.95<br>(0.95-0.95)	0.90<br>(0.90-0.91)	0.81<br>(0.80-0.81)	0.71<br>(0.70-0.71)	0.61<br>(0.61-0.61)	0.51<br>(0.50-0.51)

FH – family history, QRF– questionnaire-based risk factors, PRS– polygenic risk scores, PV– pathogenic variant

Supplementary table 4 - Participant characteristics of the UK Biobank validation cohort by age group, among those of self-reported non-White ethnicity

		Controls		Cases	
Variables	Values	N	%	N	%
Participants	N	13,908	97.6	335	2.4
Genetic risk factors					
BRCA1	Carrier	8	0.1	0	0.0
BRCA2	Carrier	22	0.2	3	0.9
PALB2	Carrier	15	0.1	2	0.6
ATM	Carrier	28	0.2	1	0.3
CHEK2	Carrier	28	0.2	0	0.0
RAD51C	Carrier	4	0.0	0	0.0
RAD51D	Carrier	6	0.0	1	0.3
BARD1	Carrier	7	0.1	2	0.6
Standardised PRS	Mean	0.669	-	0.966	-
	SD	1.0	-	1.0	-
Epidemiological risk factors					
Self-reported ethnicity	Asian	4,922	35.4	131	39.1
	Black	4,107	29.5	82	24.5
	Mixed	1,624	11.7	34	10.1
	Not known	3,255	23.4	88	26.3
Age at start of risk prediction	[40,45)	2,104	15.1	43	12.8
	[45,50)	2,991	21.5	46	13.7
	[50,55)	2,875	20.7	68	20.3
	[55,60)	2,359	17.0	67	20.0
	[60,65)	1,952	14.0	62	18.5
	[65,70]	1,627	11.7	49	14.6
	Mean	53.5	-	55.2	-
	SD	8.0	-	8.0	-
Follow up	Mean	9.8	-	5.4	-
	SD	1.1	-	2.7	-
Age at menarche	[0,11)	708	5.6	16	5.1
	[11,12)	1,584	12.5	37	11.9
	[12,13)	2,399	18.9	62	19.9
	[13,14)	2,892	22.8	72	23.1
	[14,15)	2,295	18.1	62	19.9
	[15,16)	1,521	12.0	33	10.6
	[16,17]	1,107	8.7	26	8.3
	Missing	1,203	-	23	-

Age at menopause	[0,40)	282	4.7	13	8.0
	[40,45)	686	11.4	8	4.9
	[45,50)	1,934	32.0	38	23.5
	[50,55)	2,417	40.0	77	47.5
	[55,70]	721	11.9	26	16.0
	Missing	7,868	-	173	-
Parity	[0,1)	2,416	17.9	61	18.8
	[1,2)	2,162	16.0	70	21.5
	[2,3)	4,421	32.7	90	27.7
	[3,20]	4,509	33.4	104	32.0
	Missing	400	-	10	-
Age at first live birth among parous women	[0,20)	1,578	18.1	23	12.0
	[20,25)	3,058	35.0	68	35.6
	[25,30)	2,604	29.8	63	33.0
	[30,50]	1,498	17.1	37	19.4
	Missing	2,353	-	73	-
OC use	Current	609	4.6	18	5.6
	Former	8,087	60.9	184	57.1
	Never	4,583	34.5	120	37.3
MHT use	Current C-type	889	14.7	25	15.4
	Current E-type	83	1.4	1	0.6
	Former	1,046	17.3	27	16.7
	Never	4,256	70.5	112	69.1
BMI (kg/m2)	[0,18.5)	122	0.9	2	0.6
	[18.5,25)	4,389	31.9	111	33.8
	[25,30)	4,961	36.1	103	31.4
	[30,80]	4,272	31.1	112	34.1
	Missing	164	-	7	-
	Mean	28.1	-	28.1	-
	SD	5.7	-	5.5	-
Height (cm)	[0,153)	1,978	14.4	54	16.4
	[153,160)	4,828	35.1	108	32.7
	[160,166)	4,358	31.6	106	32.1
	[166,173)	2,188	15.9	56	17.0
	[173,200]	418	3.0	6	1.8
	Missing	138	-	5	-
	Mean	159.7	-	159.4	-
	SD	6.7	-	6.7	-
Alcohol (g/day)	[0,5)	7,442	72.3	178	71.5
	[5,15)	1,908	18.5	51	20.5
	[15,25)	585	5.7	15	6.0
	[25,35)	202	2.0	3	1.2

	[35,45)	71	0.7	0	0
	[45,100]	80	0.8	2	0.8
	Missing	3,613	-	86	-

Supplementary figure 1 - Comparison of breast cancer incidences measured in the population and UK Biobank

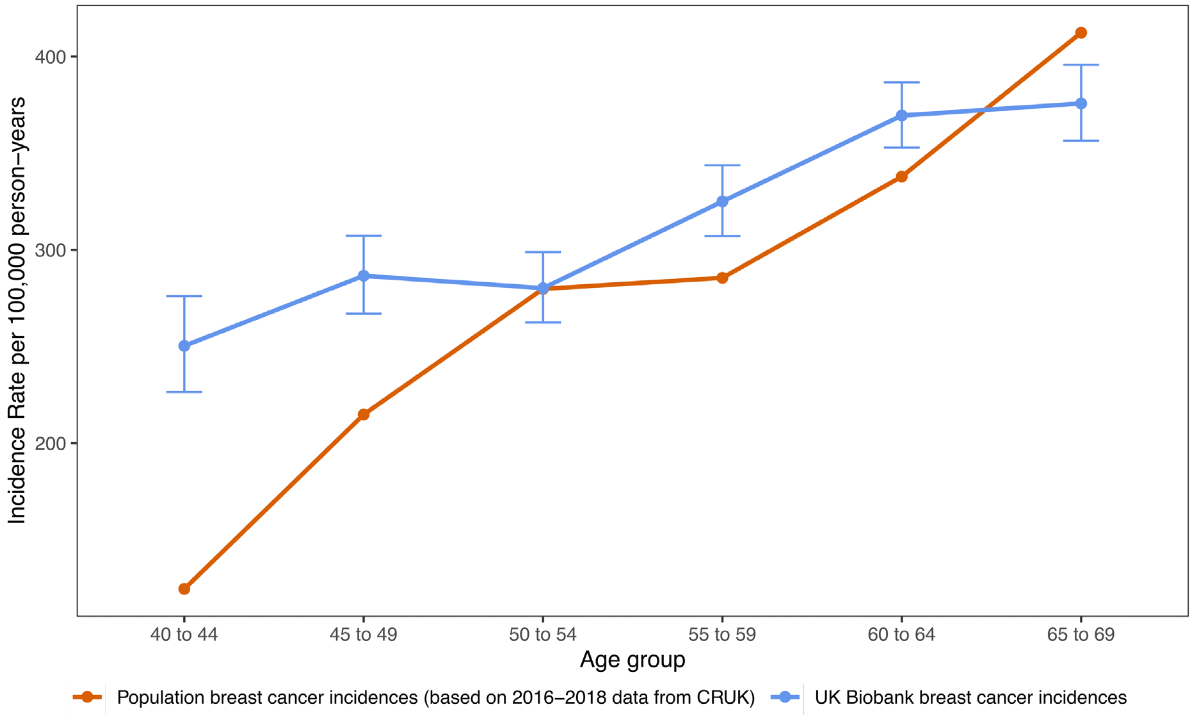

Supplementary figure 2 - Distribution of 10-year predicted risk in different BOADICEA models

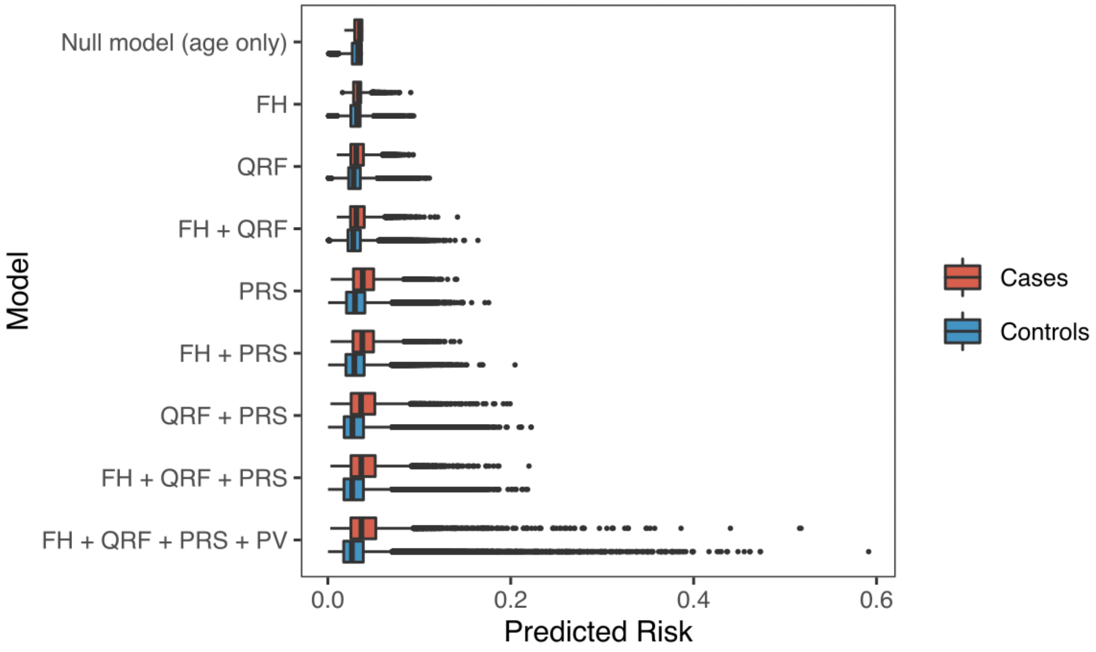

FH – family history, QRF – questionnaire-based risk factors, PRS – polygenic risk scores, PV – pathogenic variants

Supplementary figure 3 - Age-dependent absolute risk thresholds based on relative risk thresholds (solid line) and age-independent absolute risk thresholds representing 10-year absolute risk of 5.8% and 11% (dashed line), which are equivalent to relative risk of 1.6 and 3.1 at the median age of 58 years for women in the UK Biobank and age-independent absolute risk thresholds representing 10-year absolute risk of 3% and 8% (dotted line) as suggested in the NICE guideline.

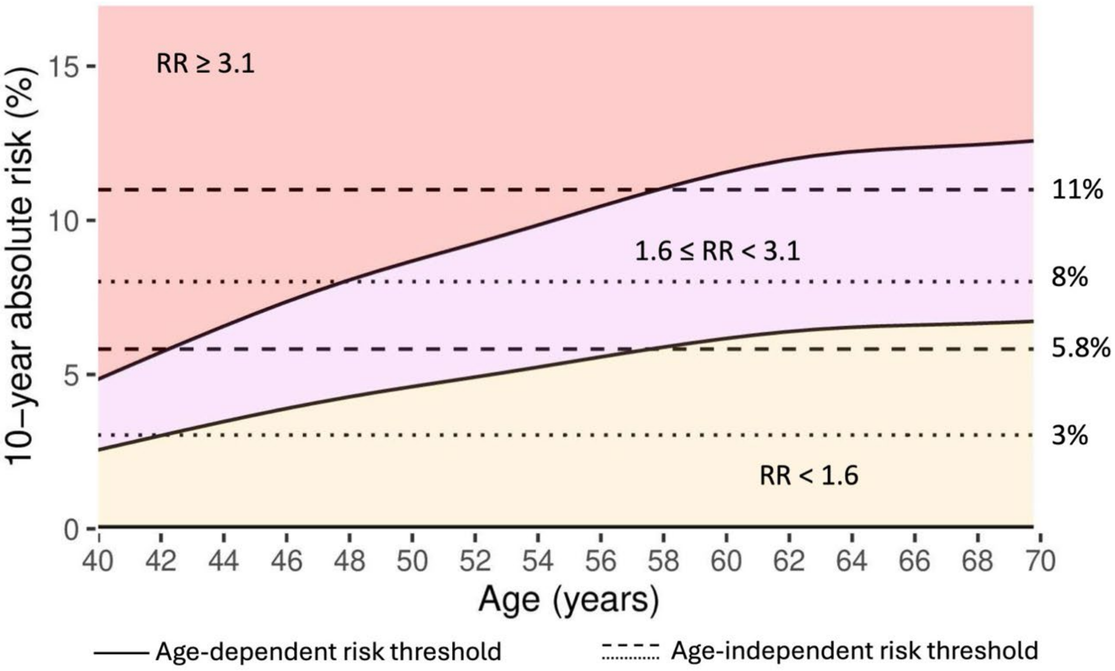

References

1. Mavaddat N, Michailidou K, Dennis J, et al. Polygenic Risk Scores for Prediction of Breast Cancer and Breast Cancer Subtypes. *Am J Hum Genet*. Published online 2019. doi:10.1016/j.ajhg.2018.11.002
